# Dietary Glycerol Monolaurate Effect on Growth Performance, Intestinal Barrier Function, and Gut Microbiota in Weaned Piglets Challenged by Lipopolysaccharide

**DOI:** 10.3390/ani15223263

**Published:** 2025-11-11

**Authors:** Huakai Wang, Ruiyu Ma, Renrong Qi, Zhen Liu, Yinghao Li, Xudong Wu, Chunfang Zhao, Qiugang Ma, Kai Zhan

**Affiliations:** 1 Anhui Provincial Key Laboratory of Livestock and Poultry Product Safety, Institute of Animal Husbandry and Veterinary Medicine, Anhui Academy of Agricultural Sciences, Hefei 230001, China; huakaiwhk@163.com (H.W.); mary0470@foxmail.com (R.M.); easona123@126.com (R.Q.); liuzhenxy2009@163.com (Z.L.); white_wxd@163.com (X.W.); 2 College of Animal Science, Anhui Science and Technology University, Chuzhou 233100, China; 13309685363@163.com (Y.L.); zhaocf@ahstu.edu.cn (C.Z.); 3 Department of Animal Nutrition and Feed Science, College of Animal Science and Technology, China Agricultural University, Beijing 100193, China

**Keywords:** glycerol monolaurate, intestinal inflammation, barrier function, gut microbiota, piglets

## Abstract

**Simple Summary:**

Weaning stress is a key factor restricting the healthy growth of piglets, among which intestinal inflammation and barrier damage triggered by lipopolysaccharide (LPS) are the core pathological links. Speaking of glycerol monolaurate (GML), as a medium and short-chain fatty acid derivative with broad-spectrum antibacterial, antiviral, and anti-inflammatory activities, its deep mechanism in alleviating intestinal inflammation has not been fully elucidated. In this study, by establishing an inflammatory model of weaned piglets induced by LPS challenge, we systematically explored the regulatory effects of GML on the growth performance, intestinal barrier integrity, and intestinal microbiota structure of piglets. The results indicated that GML could have improved the barrier function, reduced the inflammatory factors, and modulated the composition of gut microbiota in piglets challenged by LPS.

**Abstract:**

This study aimed to investigate the effects of glycerol monolaurate (GML) on the growth performance, intestinal morphology, inflammatory factors, and gut microbiota of weaned piglets challenged by lipopolysaccharide (LPS). A total of eighteen weaned piglets [Duroc × (Landrace × Yorkshire), average weight 7.54 ± 0.86 kg] were randomly assigned to the following three groups: (1) CON:basal diet; (2) LPS: basal diet + LPS challenge; and (3) LPS_GML: with 1000 mg/kg GML. On day 21, LPS or saline was injected into the piglets via intraperitoneal injection, and samples were collected after 4 h. Results showed that no differences were observed in the average daily gain (ADG), average daily feed consumption (ADFI), and G/F ratio. GML supplementation increased (*p* < 0.05) the villus height and villus height/crypt depth ratio, the protein expression of claudin-1 and occludin, the mRNA expression of *claudin-1*, *SOD*, and *GSH-Px*, while decreased (*p* < 0.05) the serum diamine oxidase (DAO) and reactive oxygen (ROS) concentrations and the mRNA expression of *IL-1β*, *IL-6*, and *IL-8* compared with the LPS group. Moreover, GML regulated LPS-induced gut microbiota dysbiosis by reshaping the composition of gut microbiota, such as increasing (*p* < 0.05) Actinobacteriota, *Lactobacillus amylovorus DSM 20531*, *Lactobacillus mucosae LM1*, and *Bifidobacterium boum*, while decreasing (*p* < 0.05) Spirochaetota. Dietary GML supplementation improved the barrier function, reduced the inflammatory factors, and modulated the composition of gut microbiota in piglets challenged by LPS.

## 1. Introduction

During early weaning, piglets face drastic environmental, psychological, and nutritional changes, which trigger severe weaning stress syndrome. This syndrome is characterized by reduced feed intake, digestive disruption, and critically, a compromised intestinal barrier function [1,2]. The core of this problem lies in the interplay between intestinal dysbiosis and mucosal immunity. The abrupt dietary shift and stress cause a rapid alteration in the gut microbiota, often leading to an overgrowth of Gram-negative bacteria such as *Escherichia coli*.

The lipopolysaccharide (LPS) derived from the outer membrane of these Gram-negative bacteria is a pivotal pathogen-associated molecular pattern that triggers innate immune responses [3,4]. Upon penetrating the compromised intestinal mucus layer, LPS binds to the Toll-like receptor 4 (TLR4) on immune cells and intestinal epithelial cells, activating the NF-κB signaling pathway. This activation leads to the massive production of pro-inflammatory cytokines, including TNF-α, IL-1β, and IL-6, resulting in local and systemic inflammation, further damaging the intestinal mucosa, increasing permeability, and ultimately causing growth retardation and increased diarrhea [5]. Therefore, strategies to mitigate LPS-induced intestinal barrier disruption are crucial for improving piglet health during weaning.

Glycerol monolaurate (GML), a monosaccharide ester recognized as safe by the US FDA, has garnered attention for its broad-spectrum antimicrobial activity, particularly against Gram-positive pathogens [6]. However, its effects on Gram-negative bacteria and, more importantly, on the host’s inflammatory response to bacterial components like LPS are less explored. Interestingly, emerging evidence suggests that GML may possess immunomodulatory properties beyond its direct antimicrobial effects. For instance, GML has been shown to inhibit the production of inflammatory cytokines in human epithelial cells challenged with staphylococcal toxins [7,8], indicating its potential to directly interfere with pro-inflammatory signaling pathways.

While dietary GML has been reported to improve gut morphology and antioxidant capacity in weaned piglets [9], its specific role in modulating the inflammatory cascade and microbial ecology during an explicit LPS challenge remains unclear. Given the central role of LPS in weaning-associated intestinal dysfunction, we hypothesized that GML would protect against LPS-induced damage by modulating the gut microbiota and attenuating the inflammatory response. Therefore, the present study aimed to investigate the effects of GML on intestinal barrier function, inflammatory status, and gut microbiota structure in weaned piglets challenged with LPS, thereby providing novel insights into its application as a nutritional intervention for weaning stress.

## 2. Materials and Methods

All animal experiments were approved by the animal ethical committee of Anhui Academy of Agriculture Science (Hefei, China; No. AAAS2025-12). All animal experiments were performed at the Animal Experiment Base of Anhui Academy of Agricultural Sciences (Hefei, China). The GML was purchased from the Shandong Binzhou GIN&ING New Material Technology Co., Ltd. (Binzhou, China). LPS (E. coli serotype 055:B5) was purchased from the Sigma-Aldrich Corporation (St. Louis, MI, USA).

### 2.1. Animals, Diets, and Experimental Design

A total of 18 [Duroc × (Landrace × Yorkshire)] weaned piglets with an average initial body weight (BW) of 7.54 ± 0.86 kg were randomly divided into three groups with six replicates per group (one piglet per replicate) based on their BW and sex. The dietary treatment included CON (a corn–soybean basal diet), LPS (a corn–soybean basal diet + LPS challenge), and GML_LPS (a corn–soybean basal diet with 1000 mg/kg GML + LPS challenge). The experiment diet (Table 1) was formulated to meet the recommended requirements of the National Research Council [10]. On day 21, the piglets were injected intraperitoneally with LPS (100 μg/kg body weight) to mimic continued intestinal damage [11], and the piglets in CON group were injected with the equal amount of saline. Each piglet was kept in a separate cage, which was equipped with a single feeding device and a single drinking fountain on one side. Piglets were housed in a room with relatively constant temperature (26–28 °C) and humidity (60 ± 10%) and had unrestricted access to feed and water throughout the experiment period (21 days).

### 2.2. Sample Collection

BW and feed consumption of each pig were measured on d 0 and 21 to determine average daily gain (ADG), average daily feed intake (ADFI), and gain-to-feed ratio (G/F). On day 21, 4 h after administering LPS, blood samples were collected from the anterior vena cava of each piglet and centrifuged at 3000× *g* and 4 °C for 15 min to obtain serum. Then, all piglets were euthanized using a captive bolt followed by exsanguination. Subsequently, a 5 cm jejunal segment was collected and fixed in a 4% paraformaldehyde. The jejunal mucosa and cecal chyme were collected, then immediately frozen in liquid nitrogen and stored at −80 °C for further analysis.

### 2.3. Intestinal Morphology in the Jejunum

Intestinal specimens fixed and preserved in a 4% paraformaldehyde were dehydrated, cleared, and embedded in paraffin using standard paraffin embedding techniques. The specimens were sectioned into 5 μm-thick slices and mounted on glass slides. The paraffin sections were then deparaffinized and stained with hematoxylin and eosin (H and E). Villus height and crypt depth were measured under a microscope using an image processing and analysis system (Version 1; Leica Imaging Systems Ltd., Cambridge, UK) at a combined magnification of 40×. At least twelve well-oriented, intact villi, and their associated crypt depths were identified and measured per section. The villus height-to-crypt depth ratio was calculated.

### 2.4. Analysis of Serum Indicators

The concentration of reactive oxygen species (ROS), D-lactate, diamine oxidase (DAO), and LPS was measured using the ELISA kit following manufacturer’s instructions (Nanjing Jiancheng Bioengineering Institute, Nanjing, China).

### 2.5. Real-Time PCR

Total RNA was extracted from the jejunal mucosa using the TRIpure reagent (Aidlab Biotechnologies Co., Ltd., Beijing, China). The quality and concentration of RNA were measured using the NanoDrop 2000 (Thermo Fisher Scientific, Waltham, MA, USA). RNA-reverse transcription and quantitative PCR were conducted with the HiScript III 1st Strand cDNA Synthesis Kit (+gDNA wiper) and ChamQ SYBR qPCR Master Mix (Vazyme Biotech Co., Ltd., Nanjing, China) following the manufacturer’s instructions. The primers for the real-time PCR are listed in Table 2. The 2^−ΔΔCT^ method was used to calculating the respective expression level of target genes. The relative mRNA expression was normalized to *GAPDH*.

### 2.6. Immunofluorescence Staining

Immunofluorescence staining was conducted as described in the previous study [12]. The primary antibodies were as follows: occludin (proteintech, Cat No. 27260-1-AP) (Wuhan, China), claudin-1 (proteintech, Cat No. 13050-1-AP), ZO-1 (abcom, Cat No. ab307799) (New Brighton, PA, USA), and β-actin (proteintech, Cat No. 66009-1-Ig). Secondary antibodies were Goat Anti-Mouse IgG (CST, Cat No. 7074P2) (Danvers, MA, USA) and Goat Anti-rabbit IgG (CST, Cat No. 91196S).

### 2.7. Gut Microbiota Analysis

Here, 16S rRNA sequencing and bioinformatics analysis were completed by referring to a previous study [13]. The primers used in this study were shown in Table 2.

### 2.8. Statistical Analysis

Data were analyzed using the SPSS software (Version 29.0). Individual piglets were treated as the experimental unit to analyze intestinal morphology, RNA level, protein level, serum indicators, and gut microbiota. For growth performance, intestinal morphology, RNA expression levels, protein levels, and serum indicators, data were analyzed by a one-way analysis of variance (ANOVA). When a significant main effect was detected, differences among the three groups (CON, LPS, GML_LPS) were further compared using Tukey’s honestly significant difference (HSD) post hoc test for multiple comparisons. The bacterial community at the level of phylum and species were analyzed by the Kruskal–Wallis rank-sum test. Data were shown as the mean ± standard error of the mean (SEM). *p* < 0.05 indicated statistical significance.

## 3. Results

### 3.1. Growth Performance

The effects of GML supplementation on growth performance in weaned piglets challenged with LPS are shown in Table 3. No differences in ADG, ADFI, and G/F were observed among the three groups.

### 3.2. Intestinal Morphology

**Figure 1:**
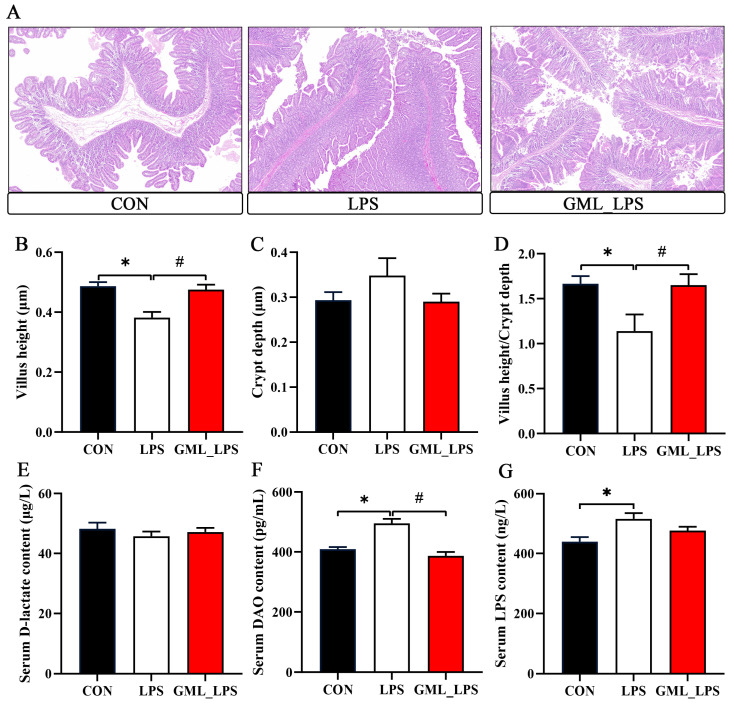
Effects of dietary GML supplementation on intestinal morphology in piglets challenged with LPS. (**A**) HE staining of piglet jejunum. Scale bar, 200 μm; (**B**–**D**) Jejunal villus height, jejunal crypt depth, and ratio of villus height to crypt depth in piglets; (**E**–**G**) Serum D-lactate, DAO, and LPS concentrations in piglets. DAO, diamine oxidase; LPS, lipopolysaccharide. * *p* < 0.05 as compared with the CON group; # *p* < 0.05 as compared with the LPS group.

Compared with the CON group, LPS challenge decreased (*p* < 0.05) villus height and villus height/crypt, while GML supplementation reversed these results (Figure 1A,B). Moreover, GML supplementation decreased (*p* < 0.05) the serum DAO concentration in piglets challenged with LPS (Figure 1F) and LPS challenge increased (*p* < 0.05) the serum LPS concentration (Figure 1G). However, LPS challenge and GML supplementation did not affect crypt depth and serum D-lactate concentration.

### 3.3. Tight Junction Protein

**Figure 2:**
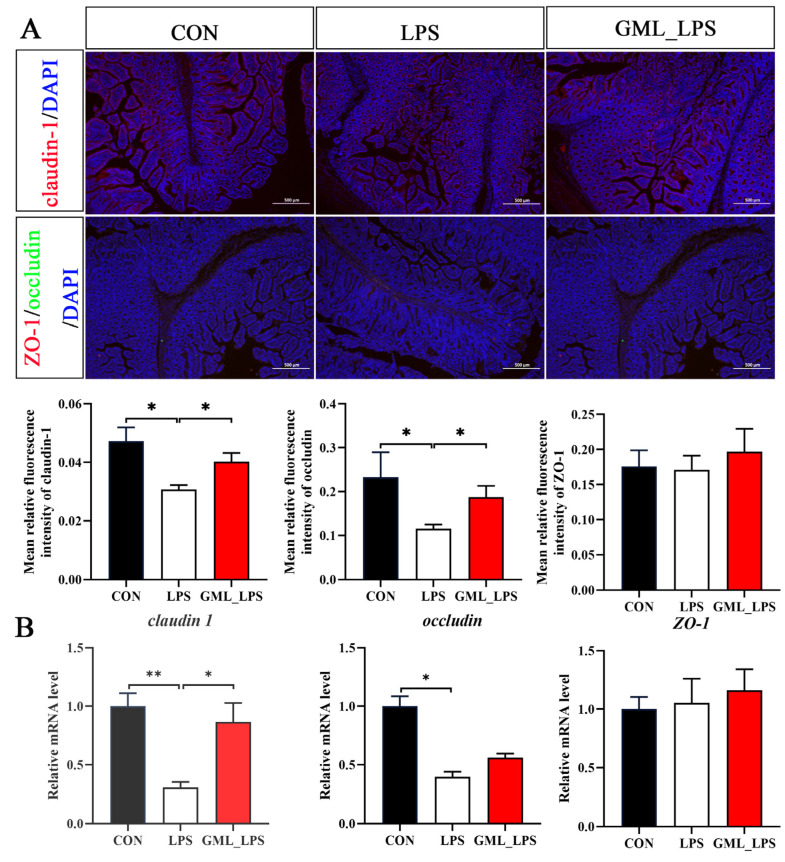
Effects of dietary GML supplementation on tight junction proteins in piglets challenged with LPS. (**A**) Typical images of immunofluorescence staining of claudin-1, occludin, and ZO-1 in the colon (500× magnification); (**B**) mRNA levels of *claudin-1*, *occludin*, and *ZO-1*. * and **: *p* < 0.05 as compared with the CON group.

As shown in Figure 2, LPS challenge decreased (*p* < 0.05) the protein expression and the mRNA expression of claudin-1 and occludin compared with the CON group. However, GML_LPS had higher (*p* < 0.05) protein expression of claudin-1 and occludin and mRNA expression of *claudin-1* compared with the LPS group.

### 3.4. Inflammatory Factors and Antioxidant Enzymes

**Figure 3:**
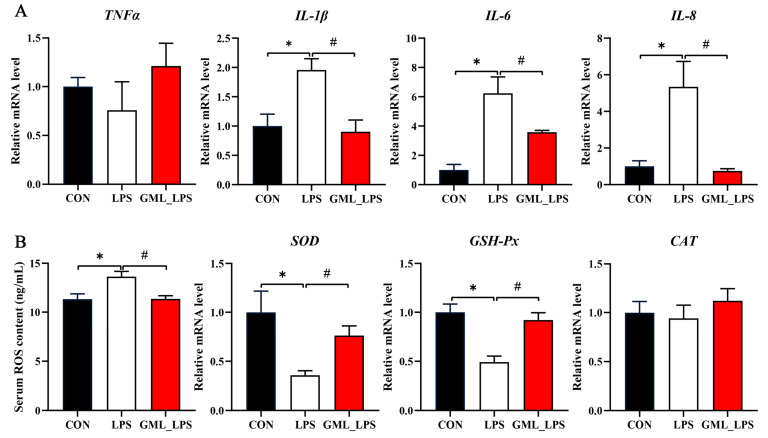
Effects of dietary GML supplementation on inflammatory factors and antioxidant enzymes in piglets challenged with LPS. (**A**) mRNA levels of *TNF-α*, *IL-1β*, *IL-6*, and *IL-8*; (**B**) Serum ROS concentration and mRNA levels of *SOD*, *GSH-Px*, and *CAT*. ROS, reactive oxygen. * *p* < 0.05 as compared with the CON group; # *p* < 0.05 as compared with the LPS group.

Compared with the CON group, LPS challenge increased (*p* < 0.05) the mRNA expression of *IL-1β*, *IL-6*, and *IL-8*, and serum ROS concentration, while decreasing (*p* < 0.05) the mRNA expression of *SOD* and *GSH-Px* (Figure 3). However, GML supplementation reversed the above results in piglets challenged with LPS.

### 3.5. Gut Microbiota Community

**Figure 4:**
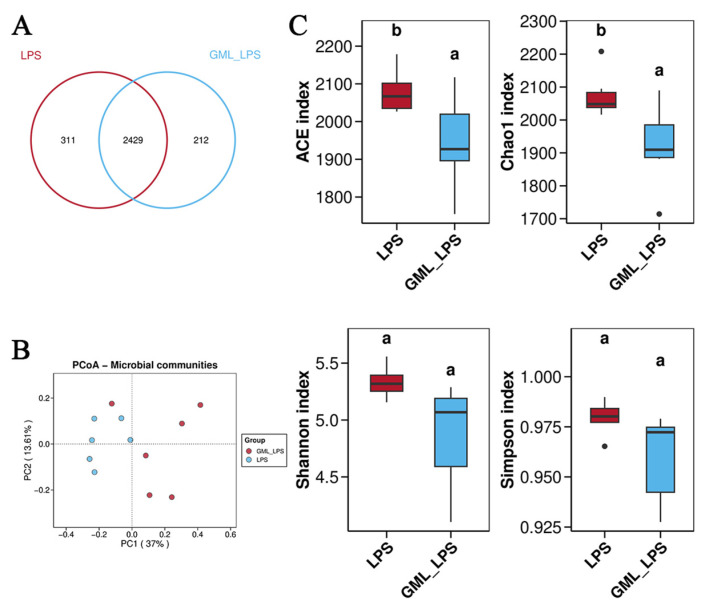
Effects of dietary GML supplementation on the α-diversity of gut microbiota in piglets challenged with LPS. (**A**) Venn diagram of OTUs; (**B**) Principal coordinate analysis (PCoA) of gut microbiota; (**C**) ACE index, Chao1 index, Shannon index, and Simpson index. ^a,b^ Different letters indicate differences between groups.

**Figure 5:**
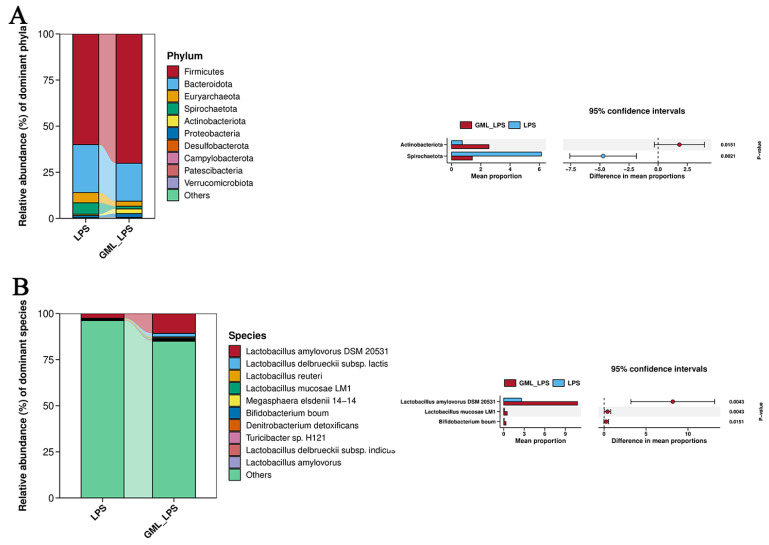
Effects of GML treatment on intestinal bacterial communities of piglets. (**A**) Abundance of the intestinal microbiota at the phylum levels; (**B**) Abundance of the intestinal microbiota at the species levels.

The Venn diagram result displayed a total of 2429 shared OTUs were present in the three groups, while LPS and GML_LPS groups contained 311 and 212 different OTUs, respectively (Figure 4A). The results of PCoA showed a distinct separation among the three groups, indicating that GML altered the gut microbiota composition in piglets (Figure 4B). Figure 4C showed that GML supplementation decreased (*p* < 0.05) the ACE index and Chao1 index. At the phylum level, Firmicutes and Bacteroidetes were the dominant phyla (Figure 5A). GML supplementation increased (*p* < 0.05) the abundance of Actinobacteriota while it decreased (*p* < 0.05) the abundance of Spirochaetota. At the species level (Figure 5B), GML supplementation increased (*p* < 0.05) the abundance of *Lactobacillus amylovorus DSM 20531*, *Lactobacillus mucosae LM1*, and *Bifidobacterium boum*.

## 4. Discussion

GML, as a green alternative to antibiotics, has the functions of emulsification, preventing feed from going moldy, and promoting the growth and health of animals [14]. A lot of research indicated that dietary GML supplementation could improve the growth performance in broilers, which may be attributed to the increased feed intake and improved intestinal health [15,16,17]. However, GML supplementation had no effect on the ADG, ADFI, and G/F ratio in piglets in the present study. In agreement with the current results, Thomas et al. showed that supplementation with 3 g/kg GML had no effect on the growth performance in pigs [18]. Additionally, Cui et al. also reported that the addition of GML to a low-protein diet could not improve the growth performance in weaned piglets [19]. Our results showed that the ADG of CON and LPS groups were 302 g/d and 299 g/d, and GML supplementation (322 g/d) increased by 6.62–7.69%, while no significant difference was found. The inconsistency of these research results may be attributed to the variations in species and the distinctions in diet types.

The intestine is the central organ for stress response, and the integrity of the intestinal barrier function is the cornerstone for ensuring the health and promoting the growth of piglets [20]. When the barrier function is impaired, a large amount of antigens floods in, which can over-activate the immune system, leading to a persistent immune-inflammatory response that inhibits the growth performance and possibly triggers the autoimmune problems [21]. Morphological indicators, such as villus height, crypt depth, and villus height/crypt depth ratio, can directly reflect the integrity of the intestinal tract [7]. In the present study, LPS challenges decreased the villus height and the villus height/crypt depth ratio, while GML supplementation reversed this result. Extensive damage and shedding of intestinal epithelial cells increase intestinal permeability, leading to the release of D-lactate and DAO into the bloodstream, with their plasma activity being positively correlated with the extent of mucosal injury [22]. GML supplementation also restored the increase in serum DAO concentration caused by LPS stimulation in this study. Moreover, tight junction proteins, such as occludin and claudin-1, play a crucial role in regulating the structural composition and permeability of the intestinal epithelium [23]. Our results show that GML supplementation could have improved the protein expression of claudin-1 and occludin and the mRNA expression of *claudin-1* in piglets challenged with LPS. These results indicated protective potential of GML in the intestinal barrier function. In agreement with the present study, previous studies showed that dietary GML improved intestinal barrier in different species and might effectively prevent bacterial endotoxins and toxic macromolecules from entering the circulatory system [19,24,25].

The interplay between intestinal inflammation and oxidative stress forms a vicious cycle that amplifies tissue damage. On one hand, the influx of inflammatory cells and the activation of pro-inflammatory signaling pathways, particularly the TLR4/NF-κB pathway, are potent generators of ROS, thereby exacerbating oxidative stress [26]. On the other hand, oxidative stress can further activate these same inflammatory pathways, creating a feed-forward loop of damage. Our findings position GML as a potential breaker of this cycle. The observed attenuation of key inflammatory cytokines (*IL-1β*, *IL-6*, and *IL-8*) at the mRNA level strongly suggests that GML’s primary action may involve the suppression of the upstream inflammatory cascade. This anti-inflammatory effect is logically consistent with the subsequent reduction in systemic ROS and the bolstering of the jejunal antioxidant defense system, as evidenced by the upregulation of *SOD* and *GSH-Px*. The findings by Kong et al. [27], which link GML to the TLR4/NF-κB pathway, provide a plausible mechanistic framework for our observations: GML may directly or indirectly inhibit this central signaling hub, leading to concurrent reductions in both inflammatory and oxidative mediators. This dual activity highlights GML’s potential advantage over interventions that target only one arm of this pathological cycle. Further research is warranted to precisely delineate the molecular targets of GML within this network.

Disorder of the microbiota is an important cause and manifestation of dysfunction of the barrier. This study demonstrated that GML could regulate the microbial community in the gut at the phylum level. The population of Actinobacteriota was higher while Spirochaetota was lesser in GML_LPS group compared with LPS group. Actinobacteriota is a group of Gram-positive bacteria with a high GC content, which plays an important role in improving growth performance, decreasing the abundance of conditioned pathogen, and regulating the intestinal immune system [28]. Change in the diversity or abundance of Spirochaetota is associated with inflammatory states, as they can adhere to mucosal surfaces, disrupt barrier function, and are linked to chronic diarrhea and intestinal inflammation [29]. A previous study has reported that GML can increase the ratio of Firmicutes to Bacteroidota [7], which contrasted with the current study, possibly due to variations in the piglets’ rearing environment and diet. The abundance of *Lactobacillus amylovorus DSM 20531*, *Lactobacillus mucosae LM1*, and *Bifidobacterium boum* was significantly increased in the GML_LPS group. *Lactobacilli* is one of the core beneficial bacteria in piglets, which plays an important role in enhancing the intestinal barrier function, regulating the immune response, and producing beneficial metabolites [30]. *Bifidobacterium* is a beneficial physiological bacterium in the gut of both humans and animals, which helps to improve digestive problems, has antibacterial and antiviral effects, reduces inflammation, enhances immunity, and has antioxidant activity [31,32,33]. Taken together, GML supplementation promoted the colonization of beneficial bacteria and inhibited the growth of harmful bacteria in piglets challenged by LPS.

Despite the promising findings, this study has certain limitations that should be considered. Firstly, the investigation focused primarily on the jejunum for the assessment of intestinal barrier function, inflammatory response, and antioxidant capacity. The effects of GML on other intestinal segments, such as the duodenum and ileum, remain to be elucidated. Secondly, while we observed significant alterations in the gut microbiota at the phylum level and for specific beneficial genera, a more comprehensive analysis, such as metagenomic sequencing, would provide deeper insights into the functional changes within the microbial community. Thirdly, the protective effects of GML were demonstrated in an acute LPS challenge model, which may not fully replicate the complexities of chronic or naturally occurring intestinal disorders in piglets. Further research under natural conditions or using different disease models is warranted to validate the practical efficacy of GML. Finally, the precise molecular mechanisms by which GML modulates this pathway and exerts its anti-inflammatory and antioxidant effects require further investigation through more targeted experimental approaches.

## 5. Conclusions

In conclusion, this experiment indicated that supplementation with GML could improve the barrier function, reduce the inflammatory factors, and modulate the composition of gut microbiota in piglets challenged by LPS.

## Figures and Tables

**Table 1:** Composition and nutrient level of basal diet.

Ingredient	%	Nutrient Level ^b^	
Corn	65.06	Digestible Energy ^b^, MJ/kg	16.20
Soybean meal 46%	14.00	Crude protein, %	19.31
Extruded soybean	6.00	Calcium, %	0.81
Soy protein concentrate	4.00	Available Phosphorus, %	0.40
Fish meal	2.00	Lysine, %	1.51
Whey powder	5.00	Methionine, %	0.43
Calcium hydrogen phosphate	1.15	Threonine, %	0.92
Limestone	1.00	Tryptophan, %	0.26
Salt	0.30		
Lysine 98.5%	0.61		
Methionine 98.5%	0.12		
Threonine	0.20		
Tryptophan	0.06		
Premix ^a^	0.50		
Total	100		

^a^ Supplied for each kilogram of the diet: vitamin A, 10,000 IU; vitamin D3, 400 lU; vitamin E, 10 mg; vitamin K3, 0.5 mg; vitamin B1, 0.5 mg; vitamin B2, 3.75 mg; vitamin B6, 2 mg; vitamin B12, 24 μg; biotin, 0.3 mg; folic acid, 3 mg; nicotinic acid, 8.75 mg; pantothenic acid, 6.25 mg; choline, 0.4 g; Fe, 96 mg; Cu, 8 mg; Mn, 40 mg; Zn, 120 mg; I, 0.3 mg; Se, 0.25 mg. ^b^ Calculated value.

**Table 2:** Primers employed in qPCR.

Gene	Accession Number	Primer Sequences (5′→3′)
*GAPDH*	XM_047787748.1	F:ACCCAGAAGACTGTGGATGG
		R:AAGCAGGGATGATGTTCTGG
*TNF-α*	NM_214022.1	F:TTGAGCATCAACCCTCTGGC
		R:ATTGGCATACCCACTCTGCC
*IL-1β*	NM_001302388.2	F:CCGCCAAGATATAACTGAC
		R:GCAGCAACCATGTACCAA
*IL-6*	NM_214399.1	F:ACCGGTCTTGTGGAGTTTCA
		R:GCATTTGTGGTGGGGTTAGG
*IL-8*	NM_213867.1	F:TTCCAAACTGGCTGTTGCCT
		R:ACAGTGGGGTCCACTCTCAA
*claudin-1*	NM_001244539.1	F:GGTGACAACATTGTGACGGC
		R:TTACCATCAAGGCACGGGTT
*occludin*	XM_005672525.3	F:TAATGGGCGTCAACCCAACA
		R:GTAGAGTCCAGTCACCGCAG
*ZO-1*	XM_047766890.1	F:GCCATCCACTCCTGCCTAT
		R:CGGGACCTGCTCATAACTTC
16S rRNA		341F:CCTACGGGNGGCWGCAG
		806R:GGACTACHVGGGTWTCTAAT

**Table 3:** Effects of dietary GML supplementation on growth performance in piglets.

Item	CON	LPS	GML_LPS	SEM	*p*-Value
BW 0 d (kg)	7.55	7.54	7.51	0.12	0.99
BW 21 d (kg)	13.90	13.82	14.27	0.42	0.91
ADG (g/d)	302	299	322	16.23	0.84
ADFI (g/d)	483	498	503	16.56	0.89
G/F ratio	1.63	1.70	1.59	0.04	0.51

SEM, standard error of the mean (n = 6); BW, body weight; ADG, average daily gain; ADFI, average daily feed intake; G/F, average daily gain/average daily feed intake.

## Data Availability

The data presented in this study are available from the corresponding author on request.

## Abbreviations

The following abbreviations are used in this manuscript:

ADFI	Average daily feed consumption
ADG	Average daily gain
DAO	Diamine oxidase
G/F	Gain-to-feed ratio
GML	Glycerol monolaurate
GRAS	Generally recognized as safe
ROS	Reactive oxygen
H&E	Hematoxylin and eosin
LPS	Lipopolysaccharide
